# Understanding the Significance and Implications of Antibody Numbering and Antigen-Binding Surface/Residue Definition

**DOI:** 10.3389/fimmu.2018.02278

**Published:** 2018-10-16

**Authors:** Mathieu Dondelinger, Patrice Filée, Eric Sauvage, Birgit Quinting, Serge Muyldermans, Moreno Galleni, Marylène S. Vandevenne

**Affiliations:** ^1^Centre d'Ingénierie des Protéines, InBios, University of Liege, Liège, Belgium; ^2^Département Biotechnologie, CER Groupe, Aye, Belgium; ^3^Centre de Recherche des Instituts Groupés, Haute Ecole Libre Mosane, Liege, Belgium; ^4^Department of Cellular and Molecular Immunology, Vrije Universiteit Brussel, Brussels, Belgium

**Keywords:** numbering scheme, complementary determining regions, antibody humanization, antigen binding residue, antibody engineering

## Abstract

Monoclonal antibodies are playing an increasing role in both human and animal health. Different strategies of protein and chemical engineering, including humanization techniques of non-human antibodies were applied successfully to optimize clinical performances of antibodies. Despite the emergence of techniques allowing the development of fully human antibodies such as transgenic Xeno-mice, antibody humanization remains a standard procedure for therapeutic antibodies. An important prerequisite for antibody humanization requires standardized numbering methods to define precisely complementary determining regions (CDR), frameworks and residues from the light and heavy chains that affect the binding affinity and/or specificity of the antibody-antigen interaction. The recently generated deep-sequencing data and the increasing number of solved three-dimensional structures of antibodies from human and non-human origins have led to the emergence of numerous databases. However, these different databases use different numbering conventions and CDR definitions. In addition, the large fluctuation of the variable chain lengths, especially in CDR3 of heavy chains (CDRH3), hardly complicates the comparison and analysis of antibody sequences and the identification of the antigen binding residues. This review compares and discusses the different numbering schemes and “CDR” definition that were established up to date. Furthermore, it summarizes concepts and strategies used for numbering residues of antibodies and CDR residues identification. Finally, it discusses the importance of specific sets of residues in the binding affinity and/or specificity of immunoglobulins.

## Introduction

In 1986, Muromonab-CD3 was the first monoclonal antibody (mAb) approved as a drug for human therapy. This murine antibody directed against the T lymphocyte CD3 complex has been widely used to prevent acute rejection in patients with organ transplants (1, 2). To date, the Food and Drug Administration (FDA) has approved 71 mAbs^1^. These antibodies are mostly used against cancers and immunological disorders (3). Furthermore, multiple mAbs were proven to be efficient in the treatment of various pathologies such as bone loss (Denosumab), hypercholesterolemia (Evolocumab) or infectious diseases (Raxibacumab, Palivizumab).

Over the last decade, with the emergence of the deep sequencing techniques, an important number of new antibody sequences have been reported. In addition, numerous 3D structures of antibodies in complex with their target antigen have been reported and have permitted statistical identification of residues that are in direct contact with the antigen or that affect the binding affinity (4–7). This has allowed approaching the molecular basis of antigen-antibody interactions. Although different bioinformatic tools, based on structural data, have been developed to predict antigenic epitopes or the interaction surface between a known antibody and its antigen (docking), *in silico* approaches are currently not able to tailor *de novo* the specificity of an antibody for a target antigen. In contrast, *in vivo* immunization techniques or selection of antibodies from combinatorial libraries by phage display are often used and were proven to be efficient to obtain specific antibodies directed against a given antigen of interest.

It is well documented that the structure of an immunoglobulin forms a Y-shaped glycoprotein (~150 kDa) that is composed of two identical heavy and two identical light chains. These heavy and light chains are each encoded by genes that have diverged from the same ancestral gene. The variable domains of the light and heavy chains are responsible for antigen binding while the constant domains communicate with other components of the immune system. Notably, besides these “standard” immunoglobulins, camelids as well as some cartilaginous fish express another type of antibody that is devoid of light chain and is referred to as heavy-chain antibody (HcAb) or immunoglobulin new antigen receptor (IgNAR), respectively. These homodimeric antibodies are able to bind to their antigen with similar affinity as conventional heterotetrameric antibodies. In all cases, the variable domains of each chain contain three hypervariable loops named complementary determining regions (CDR-1,-2, and-3). The CDRs are separated by structurally conserved regions called framework regions (FR-1,-2,-3, and-4) that form a “core” β-sheet structure displaying these loops on the surface of the variable domain. The length and composition of the CDR sequences are highly variable, especially in the CDR3. The origin of this diversity lies in the complexity of the genetic mechanisms that generate the highly variable pool of antibodies from a relatively small number of antibody genes. Variable regions are assembled from two genes (V and J, for λ and κ light chains) or three genes (V, D and J for heavy chains), following the V(D)J recombination mechanism. The joined regions are part of CDR3. Further variability in CDR3 length and sequence is introduced by the mechanisms that permit addition or deletion of nucleotides in those junctions and by somatic hypermutations in the recombined genes. The CDRs are often approximated to the paratope of the antibody that interacts with the antigen and therefore contains the antigen-binding residues. The present review will demonstrate that this definition of the paratope is an oversimplification and doesn't exactly match with the reality.

Antibody engineering methods have triggered the attention of many research groups as well as pharmaceutical companies. Antibody engineering technologies are of increasing importance in drug development and different biotherapeutics have been developed, including exploitation of antibody fragments (8), bispecific antibodies (9) or antibody-drug conjugates (10). However, despite their increasing success in drug therapy, mAbs commonly induce adverse events when injected into patients, especially chimeric molecules that contain murine or rat sequences (11) and can lead to the appearance of human anti-globulin antibodies in the serum of the patients (12–14).

To overcome these problems, different strategies have been developed that successfully reduce the immunogenicity of mAbs and therefore the risk of immune adverse events. The simplest approach to humanize a mAb consists in replacing the IgG constant regions from animal origin with the corresponding constant regions of human immunoglobulins. These, so-called, chimeric antibodies still include the entire variable regions from animal origin that are responsible for antigen binding. However, in most cases, these variable regions contain immunogenic regions that are sufficient to trigger adverse effects including anaphylaxis (14). Therefore, further humanization methods of the variable region were developed. In this context, the CDR-grafting or Specificity Determining Residue (SDR)-grafting have become widely used methods in the field. Briefly, these approaches consist in replacing murine framework regions by homologous regions from human origin (15, 16). These, so-called, “reshaped mAbs” show fewer immunogenic epitopes compared to chimeric antibodies. However, to reach a higher degree of antibody humanization, a complete and precise identification of immunogenic epitopes is required. In this context, various approaches are available that have drastically improved over the past few years, and have significantly refined humanization methods. These approaches include surface reshaping or veneering (17, 18), superhumanization (19), human string content optimization (20) or combinatorial approaches using phage-display libraries (21).

Unfortunately, even if these humanization techniques produce mAbs with reduced immunogenicity, they frequently lead to a loss in antibody affinity and/or specificity (22). In most cases, the main causes for this affinity loss are attributed to various factors such as imprecise definition of the CDR sequences (23), inappropriate choice of the human framework scaffold used for loop grafting and erroneous identification of structural corresponding residues from different species. Indeed, the antibody engineering techniques require an accurate identification of CDRs, antigen-binding residues as well as structural corresponding residues. Therefore an appropriate and standardized numbering scheme is crucial. Unfortunately, the establishment of a robust inter-species numbering convention is extremely challenging, especially given the high variability in CDR lengths and sequences.

The present review is divided into two parts. The first part describes and discusses the different numbering schemes of the variable regions established up to date. The second part compares the different CDR definitions and discusses the different residues involved in antigen-binding as well as a number of framework residues that, indirectly, affect the binding affinity of immunoglobulins. Finally, we discuss and suggest a general approach for antibody humanization. Adequate antibody numbering and annotation is of crucial importance in the field of antibody engineering and it will strongly advance monoclonal antibody-based human drug development.

## Numbering schemes of antibody variable domains

Antibody engineering methods require precise identification of the residues that have an impact on the interaction and/or affinity of the antibody for its target antigen. For example, as mentioned above, CDR-grafting aims to decrease the immunogenicity of non-human antibodies by engineering the variable regions directed against the target antigen. This method requires an accurate identification of the CDRs and therefore an adequate alignment of antibody sequences from human and non-human species. Moreover, as discussed later in this review, it has been shown that residues from the framework regions might also exert a strong impact on the antibody affinity (24). Thus, the precise identification of corresponding positions in human and animal immunoglobulin chains is essential. However, the use of different amino acid numbering schemes currently available in the literature is confusing and might lead to aberrant identification of framework and CDR residues. Therefore, it is of crucial importance to understand the different numbering schemes and, consequently, being able to compare them. The following section is dedicated to the description of the different numbering schemes that are compared and summarized in Table S1.

### Kabat numbering scheme

Over the past decades, sequencing and crystallization of antibodies resulted in significant increase of various sequence and structure databases, which made the comparison of the variable regions from human and animal immunoglobulins possible. In 1970, Kabat and Wu aligned 77 Bence-Jones protein and immunoglobulin light chain sequences in order to study the statistical variability in amino acid composition at the sequential positions of the variable antibody regions. They defined the “variability parameter” as the number of different amino acids at a given position divided by the frequency of the most occurring amino acid at that position. This analysis revealed three hypervariable regions in the variable region of the light chains. The presence of highly conserved residues was also demonstrated, such as the two cysteines that form a disulphide bridge at the inner core of the immunoglobulin domain and a tryptophan residue located immediately after CDRL1 (25). Likewise, three corresponding hypervariable regions were also identified in the variable heavy chain domain (26, 27). Kabat and Wu postulated that these hypervariable regions would cluster at one side of the folded domain to form a surface responsible for specific antigen recognition and referred to these hypervariable regions as “Complementarity Determining Regions” “CDR”-1,-2, and-3. This hypothesis was later confirmed and further investigated to distinguish antigen-contacting or conformational important residues within these CDRs (28).

In 1979, Kabat et al. were the first to propose a standardized numbering scheme for the variable regions of immunoglobulins (29). In their compilation of “Sequences of Proteins of Immunological Interest” (30), the amino acid sequences of the variable region of the light (λ, κ) and heavy chain of antibodies, as well as the variable region of T cell receptors (α, β, γ, δ) were aligned and numbered. They observed that the analyzed sequences exhibited variable lengths and that gaps and insertions could only be included at precise positions. Interestingly, the points of insertion were located inside the CDRs, except for CDRL2, but also at some positions inside the framework regions (30). In the numbering schemes, these insertions are identified and annotated with letters (e.g., 27a, 27b…). It is also noticeable that residue L10 is absent in all the λ light chains, while λ and κ chains are being coded by two different genes, located on different chromosomes. Over the last decades, the accumulation of sequences resulted in the creation of the KABATMAN database (31).

**Figure 1:**
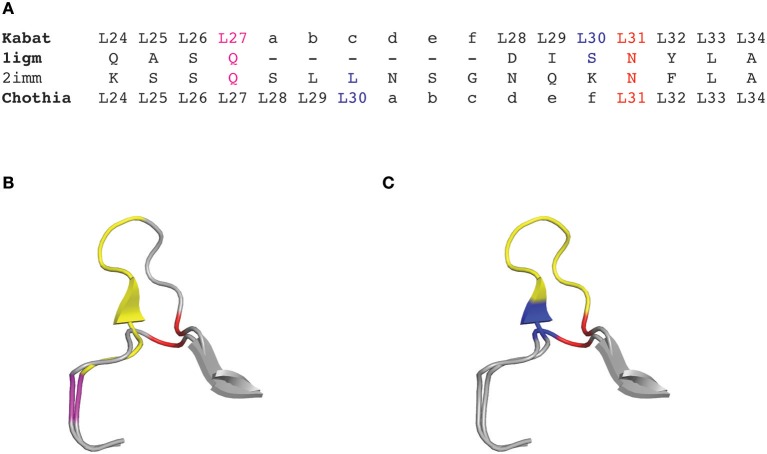
CDRL1 amino acid insertion positions according to the Kabat and Chothia numbering scheme for light chain variable domains of protein databank (PDB) structures 1igm and 2imm. **(A)** Amino acid sequence of the 1igm and 2imm CDR1L according to the Kabat or Chothia numbering scheme. The insertion of amino acids from longer loops is placed behind L27 (magenta) and denoted by ‘27a to 27f' according to Kabat's numbering; whereas they are placed behind L30 (blue) according to Chothia's numbering scheme. The structurally aligned position L31 is shown in red. **(B)** Ribbon representation of the superposition of the CDRL1 loops of 1igm and 2imm from amino acids L24 to L34. The color code is as in **(A)** and the additional amino acids of 2imm in CDRL1 (L27a to L27f) according to Kabat numbering are shown in yellow. **(C)** Same structural superposition as in **(B)** except that the yellow colored supplementary amino acids and the blue amino acid position is according to Chothia's numbering scheme.

Although the Kabat numbering scheme is often considered as the standard that is widely adopted for numbering antibody residues, it has some important limitations. Firstly, this scheme was built on the alignments of a limited number of sequences from antibodies with the most common sequence lengths. Consequently, sequences with unconventional insertions or deletions in the CDRs or in the framework regions were not included. Therefore, the original Kabat scheme ignores antibody chains of unconventional lengths, with unique insertions or deletions. However, a useful numbering tool named *ABnum*^2^ that numbers the amino acid sequences of variable domains according to a much larger and regularly updated database (Abysis^3^), takes into account insertions of variable lengths, particularly in CDR2 by adding an insertion point at position L54. The second main limitation of the Kabat scheme is that it doesn't match very well with the 3D structure of antibodies. Indeed, the hypervariable regions defined by Kabat do not exactly match with the structural antigen-binding loops. The defined insertion points in CDR-L1 (L27) and CDR-H1 (H35) do not fit with their corresponding positions in the structures (Figure 1). In other words, the corresponding residues (topologically aligned) in crystal structures in CDR-L1 and CDR-H1 don't share the same number in the Kabat numbering scheme.

### Chothia numbering scheme

In 1987, Chothia and Lesk introduced a structure-based numbering scheme for antibody variable regions. They aligned crystal structures of antibody variable regions, defined the loop structures that form the CDRs and corrected the position numbers of the insertion points inside CDRL1 and CDRH1 so that they better fit their topological positions (Figure 1) (32). Furthermore, they classified the CDR loops of heavy and light chains in a small number of conserved structures, called “canonical” classes (32–34) that will be discussed later.

**Figure 2:**
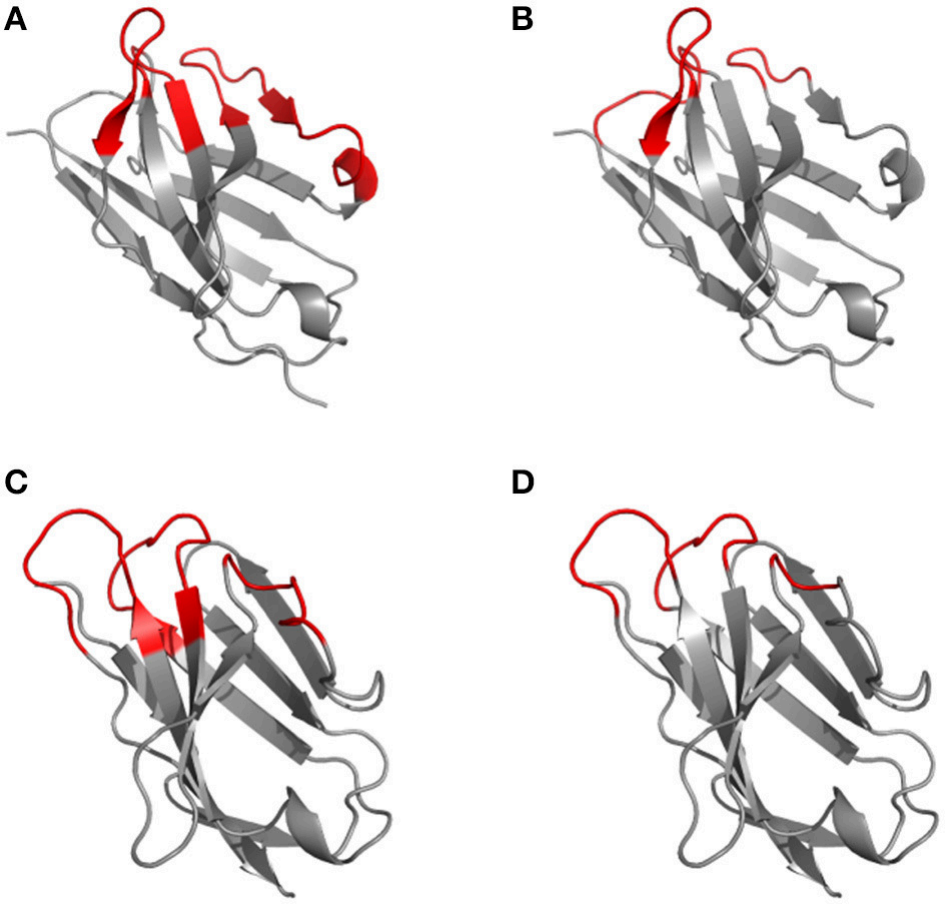
Disparity in CDR definitions according to Kabat (hypervariable regions) **(A, C)** and Chothia (structural loops) **(B, D)** shown on the 1kiq domain structures. The variable domain 3D structures of the light **(A, B)** and heavy **(C, D)** chain are represented in cartoon with the frameworks in gray and CDR highlighted in red according to Kabat **(A, C)** and Chothia **(B, D)** numbering scheme.

Based on the alignment of antibody structures, the Chothia numbering scheme shifts the point of amino acid insertion from position L27 to L30 and from position H35 to H31. It is worth mentioning that the Chothia CDR definition ensures a better correspondence to the structural loops. The loop structure of CDRH3 identified by Chothia matches well the Kabat hypervariable region. In contrast, the other loops are shorter than the hypervariable sequences defined by Kabat, except for CDRH1 which extends from H26 to H32. In any case, the CDRs defined on the hypervariable amino acids according to Kabat and based on loop topology in Chothia's nomenclature have for some CDR's a shifted location and/or comprise deviating loop lengths (Figure 2).

The Chothia numbering scheme possesses the main advantage that topologically aligned residues from different antibodies are localized at the same position number and that the Chothia CDR definition corresponds in most antibody sequences to the structural antigen-binding loops. However, confusion can also arise given the limited use of this numbering scheme compared to the Kabat or the IMGT numbering schemes (see below). Furthermore, a later study published by Chothia et al. changed the insertion point in CDR L1 from residue L30 to L31 (34). However, while investigating the conformation of the antigen-binding loops, of antibodies present in larger databases, they returned to the initial L30 position in 1997 (33). In a similar way, they initially defined an insertion point at position L93 in λ light chains (32) that was shifted to position L95 in their subsequent study (33). Finally, an important limitation of this numbering scheme is due to the use of the most common CDR sequence lengths, like the Kabat numbering scheme, and therefore the Chothia scheme ignores sequences with unconventional length. However, similarly to the Kabat numbering scheme, this system could be optimized by defining new insertion points.

### Martin numbering scheme

In a study published in 2008, Martin et al. focussed on the structural alignment of different framework regions of unconventional lengths (35). They highlighted residues that are absent in most sequences and structures and therefore the authors defined these as deletion positions. By analyzing sequences and structures, they also proposed a correction of the insertion point within the framework region 3 of the heavy chain domain from position H82 to H72. In addition and by analogy with CDRH2, they amended the position of the insertion point for the CDRL2 that locates now at position L52. Finally, they used the numbering software, ABnum, mentioned earlier and recommended a new numbering scheme that consists of the Chothia numbering system corrected by the ABnum software. Indeed, this software uses the much larger Abysis database, which integrates sequences from Kabat, IMGT, and the PDB databases. For this reason, the ABnum program defines a novel insertion/deletion position at position H6 in the Chothia and Kabat numbering schemes (6). The Martin numbering scheme corrects this point of insertion and shifts it toward position H8.

The Martin numbering scheme should be considered as the most recent version of the Chothia numbering. By analyzing sequences and structures on larger databases, it corrects insertion positions, defines new ones and highlights the location of deletions in order to fit better the topological positions of residues.

### Gelfand numbering scheme

**Figure 3:**
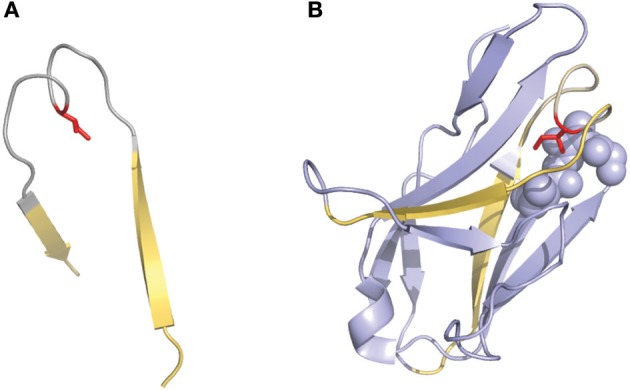
Representation of the “two-span-bridge” conformation of CDRL1 present in the 2fb4 antibody structure. The two-span-bridge is represented in gray and the bordering strands are shown in yellow **(A, B)**. **(A)** Shows the isolated loop with an Ile side chain (colored red) pointing inwards the loop structure. **(B)** The complete domain showing how the Ile penetrates deeply into the core of the light chain variable domain making hydrophobic interactions with neighboring residues organized in a pocket (purple spheres).

Another interesting numbering method that has been used in a few studies is the one described by Gelfand et al. (36–39). This numbering system results in a relatively complex nomenclature. The variable chain sequences are divided into 21 fragments termed “words,” each of these “words” matches with a secondary structure element (a strand or a loop). The strands are defined by a letter in alphabetical order (e.g., A, B, C) and the loops by two letters that corresponds to the neighboring strands (e.g., AB, BC…). However, there are two exceptions in this terminology: the three N-terminal residues of the variable chain (named OA since they are not part of the first β-strand) and the loop connecting the B and C strands, which has a ‘two span bridge' conformation with one residue deeply inserted into the structure (40) (Figure 3). This loop is divided into two words named BC and CB. This numbering system does not include gaps or deletion points but permits a precise comparison of secondary structures (loops and strands) between aligned sequences. It's also noticeable that the Gelfand definition of several loops does not exactly correspond to Chothia's definition of loops.

### IMGT numbering scheme

In 1997, Lefranc et al. introduced a new and standardized numbering system for all the protein sequences of the immunoglobulin superfamily, including variable domains from antibody light and heavy chains as well as T cell receptor chains from different species (41, 42). Their numbering scheme was based on amino acid sequence alignment of the germ-line V genes. Consequently, the amino acid sequence and numbering stops where CDR3 should start. Later on, the authors extended their numbering scheme to the entire variable domains and developed various tools to analyze the full-length sequences (43). IMGT possesses its own definitions of the framework regions (named FR-IMGT) and CDR (named CDR-IMGT).

**Figure 4:**
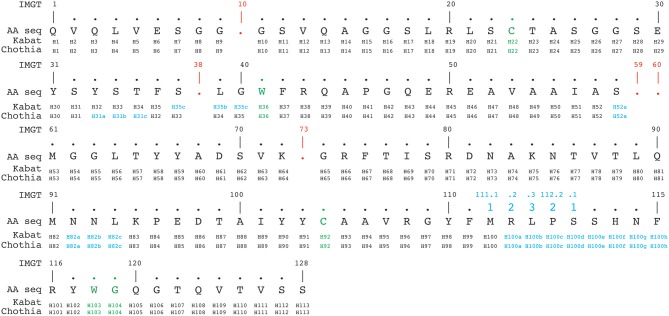
Alignment of a nanobody sequence from the PDB (3dwt) according to Kabat, Chothia, and IMGT numbering schemes. In green are the cysteines forming the conserved disulphide bond packed against a conserved tryptophan. In addition the conserved tryptophan118-glycine119 (IMGT numbering, also in green) downstream of the CDR3H demarcates the start of the framework-4 region. The IMGT numbering scheme uses gaps (indicated in red), if a residue is absent at a given position. Insertion positions are indicated in blue.

The IMGT numbering method counts residues continuously from 1 to 128 based on the germ-line V sequence alignment. Thus, it avoids the use of insertion codes, except between position 111 and 112 for CDR3-IMGT with more than 13 amino acids. Conversely, no number is attributed when a residue is missing in a particular sequence. For example, in a 6 amino acid long CDR1-IMGT, residue #27 is followed by residue #34 (and residue numbers #28–#33 are absent). An example of alignment according to Kabat, Chothia and IMGT numbering schemes is shown in Figure 4.

IMGT is the primary reference in immunogenetics and immuno-informatics. Its conventions, including its amino acid numbering method, have been recognized and are currently used by the World Health Organization—International Union of Immunological Societies. This numbering method has the main advantage that it is based on alignments of sequences from a complete reference gene database (44, 45) including the whole immunoglobulin superfamily. This has led to the development of highly useful tools. For example, amino acid alignment and numbering can be performed by the IMGT/DomainGapAlign (46). This tool also enables to analyse sequence domain polymorphisms by identifying the corresponding VDJ genes coding for the variable region. It is coupled with another interesting application known as IMGT- “Collier de Perles” (47) that allows to visualize at a glance the position of the amino acids in a 2D representation, and also to delineate easily FR-IMGTs and CDR-IMGTs.

However, due to the continuous numbering of the amino acids along the sequence, the IMGT numbering scheme does not allow an intuitive visualization of insertion positions, even for the most common ones. For the same reason, this numbering scheme is less flexible. Indeed, while in the Kabat and Chothia numbering systems, positions of amino acid insertion points are easily incorporated; it is more difficult to adapt the IMGT scheme for potential sequences with new amino acid insertions. It has to be noted that IMGT places all such insertions at the end of the CDR, which doesn't correlate with the antibody structure. However, this problem has been corrected in the later V-Quest software that places insertions in the middle of the CDR-IMGTs, which matches better with the available structural data (48).

### Honneger's numbering scheme (AHo's)

**Figure 5:**
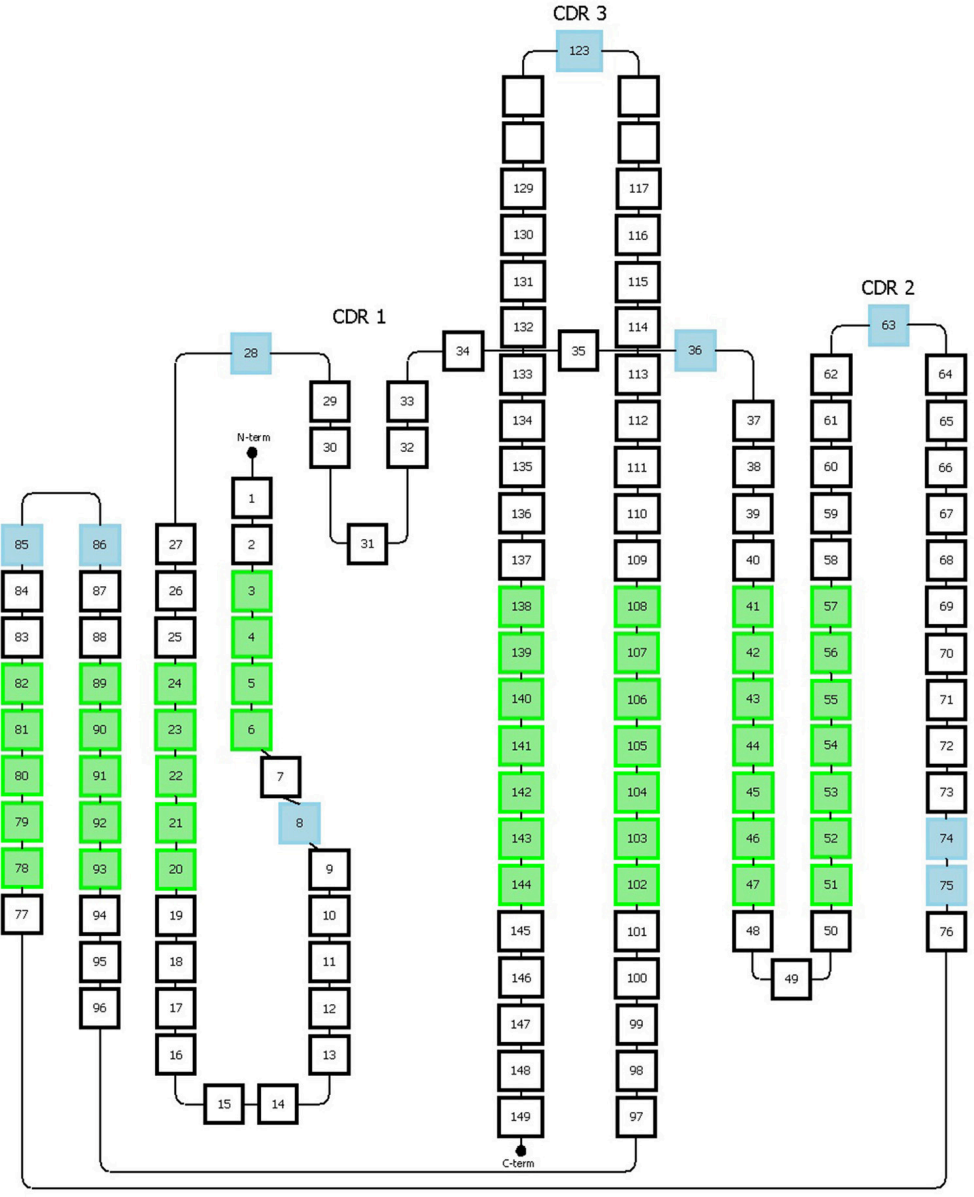
Representation of the Honneger numbering scheme. The amino acid positions that accommodate gaps are indicated in blue. The green positions correspond to the structurally conserved residues whose Cα positions were used for structural superposition. This figure has been adapted from the study published by Honneger et al. (49).

The Honegger scheme numbers the variable domains of the immunoglobulin superfamily in a homogenized format. This system is based on structural alignments of the 3D structures of the immunoglobulin variable regions covering the observed length variation. It allows to define structurally conserved Cα positions and therefore deduces appropriate framework regions and CDR lengths (Figure 5) (49).

The Honegger numbering scheme (AHo's) also defines conserved residues (C23, W43, C106, G140) and gaps on specific positions (#27-28, #36, #63, #123). The CDR1 has a “two span bridge” conformation created by a conserved hydrophobic residue at position #31 which is deeply inserted into the structure and therefore divides the loop into two distinct parts (40). The Honegger scheme describes two gap regions located onto these two parts, one located in the first part (#27 and 28) and the other one located in the second part (#36). This convention respects the variability of insertions present on both sides of the loop. Furthermore, two other insertion points are located, respectively, at position #74 and #75 to reflect the shorter C-terminal branch of the CDR2 loop exhibited by T cell receptor α. Additional gap positions are placed in the middle of the CDR-2 and-3 loops (Figure 5). From further structure analysis, they proposed to shift the insertion gap in V_κ_ chains initially located in position L10 to L8.

The main advantage of the Honegger numbering system is that it is based on structural alignments and therefore it matches better to antibody 3D structures features, in a similar manner to the Chothia numbering scheme. In addition, as mentioned for the IMGT scheme, AHo's is well-suited for the numbering of all proteins from the immunoglobulin superfamily by including two gaps into CDR 1 and 2. However, similarly to the IMGT scheme, the AHo's can skip some numbers in the sequential residue numbering which can be puzzling when analyzing the sequence numbering. This numbering scheme is also less flexible and adaptable to include immunoglobulins with new or larger insertions. Although the observed length variability was covered, it is possible that new insertion positions/lengths could be found by taking into account a larger number of structures. Furthermore, the most structurally conserved positions were obtained from only 28 different structures. Likewise, a better precision in defining the framework regions could be reached by adapting the scheme specifically to the variable region of a specific type of immunoglobulin (e.g., antibody).

Finally, the sequence numbering using the aHo's can be submitted to the PyigClassify server^4^. However, this server doesn't seem to take into account the two insertion positions defined in the original paper (49).

## Finding your way among the CDRs definitions

### CDR definitions and antigen binding residues

CDRs are commonly considered as structured loops that are involved in antigen binding and exhibiting a hyper-variable amino acid composition. However, defining a CDR based on antibody amino acid sequences can be complicated. Indeed, the different numbering schemes presented in this review utilize different definitions of CDR lengths. In addition, as shown earlier, the Kabat (and IMGT) CDR definitions are based on sequence alignments while the Chothia CDR definition better reflects the loop structure in antibodies' 3D architecture. This lack of agreement in defining precisely the CDR lengths and positions is somehow unexpected since these regions were shown to be responsible for the antigen-binding activity already a long time ago (16). In order to address the disparity in CDR definitions, several authors have taken into account the different possible lengths for defining CDRs sequences. For example, North et al. use longer sequences in their recent structural analysis of the conformations of the CDR loops (50).

Furthermore, a high binding affinity reflects a very stable antibody-antigen complex. This is accomplished by multiple non-covalent bonds between amino acid residues of the paratope and the epitope. However, it has been shown that only 20 to 33 % of the amino acids within the CDRs make direct contact with the antigen (51). These residues, named “Specificity Determining Residues (SDR)”, were first described by Padlan et al. (52). Their results show that these SDRs are involved in the interaction with the antigen and, in most cases, match with the most variable positions present in the CDRs. Using this SDR concept, MacCallum and co-workers suggested a new method to define the CDRs and re-named the SDRs “contact residues”. They also suggested that contact residues are more often located in the center of the paratope and, as Chothia mentioned before, non-contacting residues play a role in shaping the conformation of the CDR loops and therefore orientate the contact residues optimally for efficient and specific antigen binding. Finally, their study showed that small antigens tend to bind a paratope with a more concave interface (6), whereas long-convex-shaped paratopes will be better suited to bind enzyme active sites and block their catalytic activity. In this context, the use of nanobodies or bovine antibodies that exhibit an extended length of CDR3 are particularly interesting (53, 54).

Similarly, Ofran et al. used a multiple structural alignment approach to identify the antigen binding residues of the variable regions (55). They revealed that the antigen binding residues show a particular amino acid composition, as previously suggested by other groups (25, 56). In particular, tryptophan and tyrosine residues are highly over-represented, whereas almost all other residues are under-represented in all CDRs. Moreover, they showed that conventional classical CDRs identification (Kabat, Chothia, IMGT) could miss about 20% of the antigen binding residues and suggested a new method for the identification of the regions containing these antigen-binding residues. They named these regions Antigen Binding Regions (ABRs) that can be identified using the Paratome^5^ online tool. This server identifies ABR by comparing the antibody sequence with a set of antibody–antigen structural complexes (4, 55, 57). Another useful alternative tool is proABC (http://circe.med.uniroma1.it/proABC/). This software estimates the probabilities for each residue to form an interaction with the antigen (58).

Another interesting approach was offered by Robin et al. (5) studying the binding free energy of antibody-antigen complexes using a computational alanine scanning method. They showed that 80% of the binding free energy in the studied complexes is clustered on a very limited number of interacting residues (between 4 and 13). They highlighted 30 positions having major contribution to the binding free energy, 27 of them being located within the CDRs (using the Kabat definition) and the remaining 3 in the framework regions. All these positions are occupied by a restricted panel of amino acids (Y, G, S, W, D, N) where aromatic residues are the major contributors to the binding free energy. Based on the identity of these residues, the nature of the antigen-antibody interaction can be predicted and will be discussed later (see discussion section). In their analysis, CDRH2 and CDRH3 include most of these residues while CDRL2 does not contribute at all in more than half of the investigated complexes. Finally, they show that the Ab-Ag complex formation involves between 3 and 6 CDRs. They also confirmed their computational analysis using experimental data from case studies (5).

Interestingly, a simple set of rules for Kabat and Chothia CDR identification has been defined by Martin and implemented in the ANTICALIGN software (59, 60).

**Figure 6:**
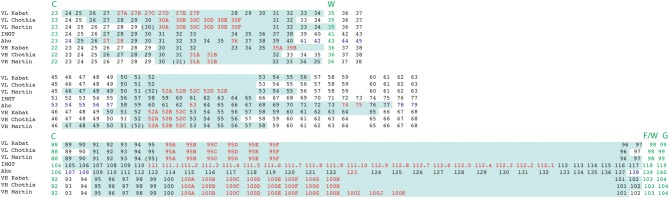
Alignment of residue positions of the CDRs within the light and heavy chain variable domains showing the disparity in the CDR definition according to the different numbering schemes. The CDRs are highlighted in pale green. Amino acid insertion positions are indicated in red. In the Aho numbering system the structurally conserved positions are colored in purple. For Martin's CDR definition, the CDRs correspond to the antigen contact residues. Conserved residues surrounding CDR 1 and 3 are indicated in green.

Figure 6 illustrates the disparity in the CDR definitions. This comparative alignment shows that CDRs defined with the classical CDR definition (Kabat, Chothia, IMGT) should be considered only as an approximation of the paratope. According to all the concepts and observations described above, a residue should be considered as part of the paratope if: (i) it is in close contact with the antigen and/or; (ii) it significantly and specifically contributes to the negative Gibbs energy change occurring upon antibody-antigen complex formation. Also, it should be noted that: (i) some contacting residues may contribute minimally to the binding free energy and even disfavor the complex formation and; (ii) that a residue energetically important for binding to the cognate antigen may not be important for the difference in affinity between cognate and non-cognate antigens and, finally; (iii) a residue crucial for antigen recognition may not be important for binding free energy. Indeed, the paratope and its corresponding epitope possess shape and chemical complementary surfaces. This shape complementarity involves mainly aromatic residues (W, F, Y) establishing van der Waals (π-π) or other hydrophobic interactions. In contrast, stable Ag-Ab complexes, characterized with low dissociation rate constants, are mostly attributed to electrostatic interactions or hydrogen bonds involving charged or polar side chain residues. Importantly, many residues included within the CDRs are not directly in contact with the antigen, but may also play an important role in the Ag-Ab interaction by upholding the optimal conformation and orientation of nearby interacting residues (33, 61).

### Structural classification of the CDRs

Ideally, an adequate definition of the CDRs should include all the residues that form the surface of the paratope and that interact with the epitope. A structural analysis of the CDRs is therefore important. Chothia's group classified the CDR loops of heavy and light chains in a small number of conserved structures, called “canonical” classes (32–34). This classification system indicates that the CDRs of the light chain (CDR L1, L2, L3) and the first two CDRs of the heavy chain (CDR H1, H2) adopt only a few different structures. These structures seem conditioned by the sequence length and presence of key amino acids at hallmark positions. The authors identified that only very few conserved residues (13 and 7 in the light and heavy chains, respectively) found within the CDR and FR regions are responsible for the conformations of the CDRs. However, controversially, it seems that there is no obvious correlation between the germ-line sequence of a CDR and its canonical class (62), most likely because small sequence differences, including in the framework regions, can impact the conformation of the CDRs (63). Beside Chothia's works, several other studies have enhanced the structural clustering of these CDRs and the identification of key residues impacting their conformations (50, 62, 64–66), including the highly variable CDRH3 (67–69). An interesting study published by De Genst et al. illustrates the relation between sequence and structure of CDRs. Briefly, they showed that the part of the CDR3 loop encoded by the same D gene in two nanobodies adopts an identical structure and targets the same epitope on the antigen (70).

In conjunction, these studies suggest that the structural classification of the CDRs based on structure prediction from the CDR sequences can be a very useful tool for antibody engineering. A few online tools are available for this purpose (71, 72)^6^,^7^. However, it is important to keep in mind that residues from the framework regions can also influence the CDR conformation as regularly discussed in this review.

## Influence of non-CDR residues on the antibody binding affinity

It is now well-established and documented that non-CDRs residues may play an important role in the binding affinity of the antibody to its antigen (73), either by making direct contact with the antigen (4, 61), by affecting the stability or flexibility of the antibody or its antigen-binding loops (74), or by structuring the CDR loop itself (75). Indeed, the residues from the framework regions can modulate the conformation of CDRs and therefore affect the binding affinity. These residues were defined and named “Vernier zone residues” and included amino acids located in the framework regions just in the vicinity of the CDR loops (75). Finally, non-CDR residues that influence the light and heavy chain variable domain packing and orientation are also critical for the antigen-binding affinity and, surprisingly, were often ignored or neglected. Therefore, the amino acids of the Vernier zone are incomplete and should be extended to include all the residues located in the frameworks impacting the affinity of the antibody. The next paragraph focuses on the residues located at the interface between the V_H_ and V_L_ that alter the packing of these domains and consequently, influence the topography of the paratope.

**Figure 7:**
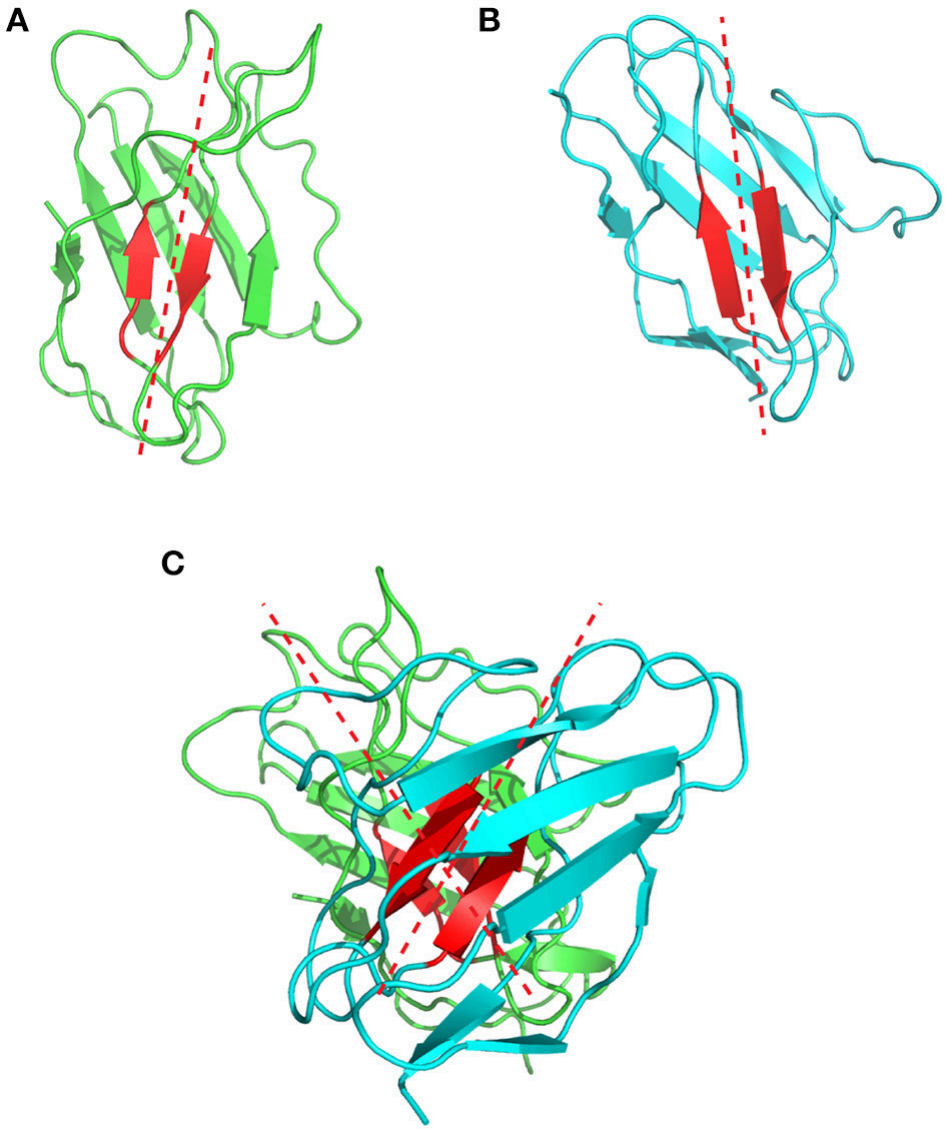
Representation of the V_H_/V_L_ packing angle in the 1bgx structure. **(A)** Shows the ribbon structure of the V_H_ and V_L_ in green and cyan, respectively. The conserved residues used to define the packing angle are shown in red. The regression lines, fitted on the Cα atoms of these residues are shown in red dashed lines. **(B)** Shows the heavy chain variable domain of the 1bgx structure and **(C)** shows the light chain variable domain of the 1bgx structure.

Chothia et al. were the first to describe and analyse the packing of the V_L_ and V_H_ domains (76). They highlighted the presence of aromatic side chains that are involved in this interface, but their analysis relied on only three antibody structures available at that time. In 2010, Abhinandan and Martin made a more comprehensive analysis of the diversity between V_H_ and V_L_ packing angles including 567 antibody crystal structures. They developed a method to predict the packing/orientation of the heavy and light chain variable domains based on the presence of specific amino acids located at the interface between these two domains. In practice, they defined Cα atoms from structurally conserved residues located at the interface between the V_H_ and V_L_ domains. These atoms were then used to fit two regression lines, one on the light and one on the heavy chain. The packing angle value corresponds to the angle formed by these two fitted vectors (Figure 7) and varies significantly from one antibody to another (i.e., −31.0° to −60.8°) with a mean of −45.6°. Their results suggest that the Chothia's loop residues have only little influence on this angle. In contrast, the authors identified 13 residues on the V_H_/V_L_ interface that influence this packing angle (77). A web tool named PAPS (Packing Angle Prediction Server) is available online for the prediction of the packing angle and is based on sequence homology^8^.

More recently, Dunbar et al. (78) further characterized the orientation of the variable domains. Indeed, one particular angle might reflect more than one single possible orientation of the V_H_ and V_L_. Therefore, they developed a new method to describe more precisely the V_H_/V_L_ orientations by defining 5 different angles and one distance. They also investigated positions in the V_H_/V_L_ interface and the residue identities influencing these different angles and distance. They concluded that a particular residue at a specific position might lead to more than one specific V_H_/V_L_ angle. They generated a new software named AB Angle that is available online^9^, to measure the different angles from PDB structures (78).

This V_H_/V_L_ angulation influences also the relative position of the CDRs and, consequently, the shape of the paratope. This parameter can therefore have a strong impact on the binding affinity. Indeed, the binding energy between two atoms is a function of their distance following the Lennard-Jones relation. A difference of a few Angstroms can strongly affect the value of the binding free energy. Using right triangle simple trigonometry and assuming a variable region length of 37 Å, a difference of 1° between the V_L_/V_H_ domains causes a displacement of the atoms exposed on the CDR surfaces by about 0.6 Å. The choice of the framework regions for humanization by the CDR-grafting technique is therefore of crucial importance to maintain affinity. For example, Nakanishi et al. showed a severe affinity loss of a humanized antibody and restored the original affinity by performing two mutations at the VH/VL interface (79). Similarly, Bujotzek et al. performed antibody humanizations by selecting human frameworks based on the predicted V_H_/V_L_ orientation and revealed a correlation between similar angles and affinity of humanized antibodies (80).

This concept of packing angles is a critical aspect of antibody-antigen interaction and the residues that modulate the V_H_/V_L_ orientation have therefore to be considered as elements that introduce further diversity in the paratopes. Framework residues affecting significantly the affinity are listed in Table S2.

## Discussion: importance of amino acid numbering and CDR definitions in antibody humanization

To achieve a high affinity binding, the paratope and its corresponding epitope must have large shape complementary surfaces and, in addition, the contacting residues must establish interactions that stabilize the complex. The parameters that influence the shape diversity of the paratope are essentially the CDR lengths and conformations (canonical classes) (32, 56, 64, 81), their relative orientations (77–79, 82) and the hydration shell (solvation) of the binding interface (83). The binding of the two complementary surfaces is mostly driven by aromatic residues that establish van der Waals and hydrophobic interactions while the strengthening of the complex involves rather electrostatic interactions and hydrogen bonds established between side chains of adequately positioned charged and polar residues.

**Figure 8:**
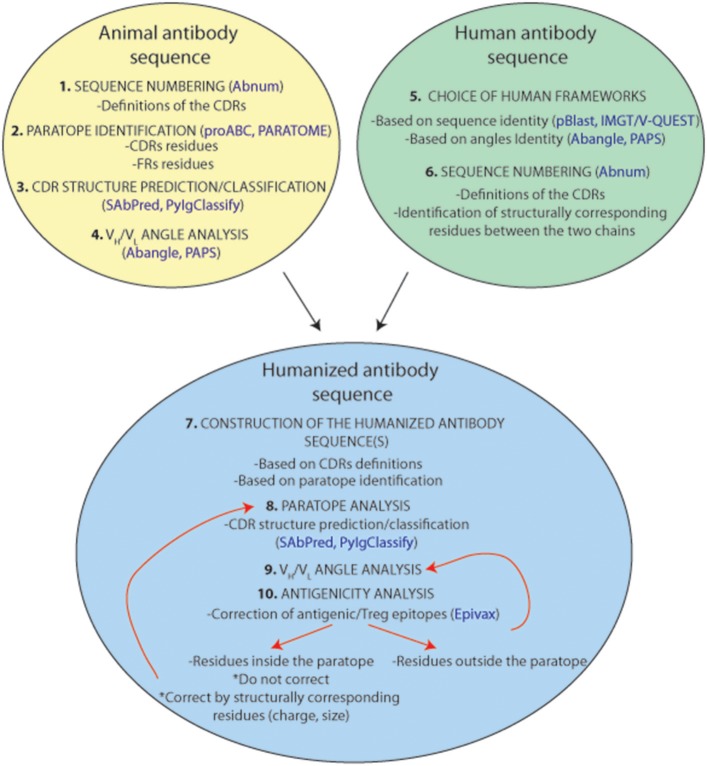
Representation of a standardized humanization protocol. Prediction tools are indicated in blue.

An antibody humanization experiment attempts to reconstitute the original paratope-epitope interactions, in most cases, by grafting the CDRs of a non-human antibody to a human antibody scaffold. This CDR-grafting or reshaping method is often based on a simplified view of antigen-antibody interaction that reduces the paratope to the 6 CDRs of the antibody. Although, there is no general protocol to perform an antibody humanization, since it is always a case-to-case experiment, this section attempts to provide guidance in such an exercise. Figure 8 summarizes and suggests a standardized protocol to humanize antibodies from animal origins. Firstly, we need to identify the CDRs within the loop of the donor antibody from animal origin. Under the simplified assumption that the paratope corresponds to the CDRs, it is recommended using the Chothia's CDR definition as they correlate very well with the structural loops present in the variable regions. A few online software tools are available for CDR structure prediction^10^,^11^ (72, 84) and classification^12^ (71). However, it is always best to choose the broadest CDR definition to ensure that all residues constituting the paratope will be included. Secondly, as discussed repeatedly in this review, residues outside the classical CDR definitions can also be part of the actual paratope. Different studies can help to identify these residues, but unfortunately, only one bioinformatical tool (Paratome) is currently available. Finally, the relative orientation of the CDRs is also critical to reconstruct the paratope surface and to position adequately its antigen interacting residues. Hence, it is essential to choose the most appropriate human V_H_ and V_L_ framework scaffolds. Selecting this human antibody scaffold is probably as important as the definition of the CDRs. The chosen human framework scaffolds should exhibit the closest V_H_/V_L_ angles compared to those in the animal antibody for a correct positioning of the CDRs in the reshaped construct. In this context, different amino acid positions have been highlighted for their role in the V_H_/V_L_ angulation and various angle prediction software tools are currently available. Thus, a reshaping effort to humanize antibodies should be an effective “paratope grafting” experiment rather than a “CDR-grafting.”

Noticeably, for all these purposes, it is important to align the antibody sequences correctly and to identify precisely the residues with superimposed positions in chains of different origins. Therefore, the handling of the same and an adequate numbering scheme that attributes an identical number of residues (including fixed possible residue insertion points) to occupy the same structural positions in the immunoglobulin chains forms a prerequisite for all antibody engineering tasks. In this respect, the enhanced Chothia's (Martin's) numbering system is a bit easier to use since it identifies precisely insertion points but, of course, this choice is quite subjective.

Another critical point is that the humanized antibodies should not induce any adverse immune reaction in the patient. Therefore, prediction of potential immunogenic epitopes in the protein sequence should be performed. Briefly, this adverse immune response takes place after internalization of the antibodies by the antigen presenting cells. Antibodies are digested in oligopeptides that bind to HLA-DR, HLA-DQ, or HLA-DP molecules. These membrane protein complexes bind specific oligopeptides and present them to lymphocytes T helpers that activate the immune response. Nowadays, databanks reporting the polymorphisms of these MHC-II molecule alleles are available to predict the oligopeptide binding potential (85–87). One of these is the Epivax web tool (88) that consists in scanning a protein sequence for identifying putative T cell epitopes. Identified antigenic sequences are predicted to bind to HLA-II DR (or other HLA-II isotypes) proteins but the program can also identify Treg epitopes that inhibit the immunity response. This software offers to mutate one or more amino acids in order to reduce the immunogenicity of a protein sequence. Sequence immunogenicity is calculated and presented in an “Epimatrix score” allowing to predict the immunogenicity of a given amino acid sequence (89). Highlighting important residues (for binding affinity/specificity, angulation, etc.) that shouldn't be mutated could improve this software. It is important to realize, that in the case of antibodies, conflicts may arise between maintaining a high affinity and a low immunogenicity. This is one of the reasons why antibody humanization remains challenging.

## Conclusion

This review describes the different amino acid numbering systems and CDR definitions that are currently available and it highlights the importance of standardized numbering system for antibody engineering strategies, especially for antibody humanization tasks. Indeed, an effective amino acid numbering system should be able to assign the same number of residues to structurally aligned positions in antibodies from different species. Although several numbering tools based on ever growing databases are available online, it is recommended to compare the different numbering systems as inaccuracies are still possible, especially for variable antibody domains with unconventional lengths.

Furthermore, the different CDR definitions and other concepts, such as contact residues or antigen binding residues, have been reviewed. In the context of antibody humanization methods, paratope has been very often limited to the CDRs. This approximation is useful as long as it permits the CDR-grafting method to be an easy and generalizable tool for humanization. In contrast, grafting contacting residues (even these outside the CDRs) and/or residues having an impact on the binding free energy would allow a better reconstitution of the paratope. However, this approach is less convenient in antibody humanization efforts because of the discontinuous nature of the paratope combined with the experimental approaches that require the precise determination of these residues. Moreover, different studies that have analyzed the angle between the light and heavy chain variable regions have been described. In CDR-grafting or other antibody humanization methods, residues affecting the V_L_/V_H_ packing angles should be considered in order to restore full binding affinity. Finally, all of these concepts that are crucial for the humanization of antibodies should be included in the humanization process.

In summary, a precise identification of the paratope established using an appropriate amino acid numbering scheme is necessary to engineer humanized antibodies with high affinity, stability and low immunogenicity. Designing a fully functional paratope on another framework should not only be restricted to grafting the antigen contacting and interacting residues, but should also include the amino acids that assist in fixating the antigen-binding loops and the residues at positions that affect the relative orientation of the paired V_L_/V_H_. All these residues have to occupy identical positions in the 3D structure.

## Author contributions

MD and MV wrote the manuscript. PF, ES, BQ, SM, MG gave regular intellectual inputs and proofread the manuscript.

### Conflict of interest statement

The authors declare that the research was conducted in the absence of any commercial or financial relationships that could be construed as a potential conflict of interest.

## Footnotes

^1^http://www.actip.org/products/monoclonal-antibodies-approved-by-the-ema-and-fda-for-therapeutic-use/

^2^http://www.bioinf.org.uk/abs/abnum/

^3^http://www.bioinf.org.uk/abysis/

^4^http://dunbrack2.fccc.edu/PyIgClassify/User/UserSequences.aspx

^5^http://ofranservices.biu.ac.il/site/services/paratome/

^6^http://dunbrack2.fccc.edu/pyigclassify/

^7^http://opig.stats.ox.ac.uk/webapps/sabdab-sabpred/WelcomeSAbPred.php

^8^http://www.bioinf.org.uk/abs/paps/

^9^http://www.stats.ox.ac.uk/~dunbar/abangle/

^10^http://opig.stats.ox.ac.uk/webapps/sabdab-sabpred/WelcomeSAbPred.php

^11^http://rosie.rosettacommons.org/antibody

^12^http://dunbrack2.fccc.edu/pyigclassify/

## Supplementary material

Table S1 Alignment of the numbering systems described by Kabat, Chothia, Martin (enhenced Chothia), Gelfand, IMGT and Honegger. VL states for variable domains of light chains, VH for variable domains of heavy chains. IMGT and Honegger (Aho) numbering systems do not take into account the nature (heavy or light) of the chain. Insertion positions are highlighted in yellow. In the Aho numbering scheme, these yellow residues correspond to the gap positions. Deletion positions are in brackets (residue L 10 in lambda light chains, Martin numbering scheme). CDR definitions are indicated in orange and framework definitions in blue (IMGT and Aho schemes). The Gelfand numbering scheme is divided in words (boxes) with the numbering below. For the Aho numbering scheme, structurally conserved position are highlighted in green. Conserved amino acids are indicated below the IMGT numbering scheme (Cys 23, Trp 41, Leu 89, Cys 104, Phe/Trp 118, Gly 119).

Table S2 Alignment of the numbering systems and CDR definitions described by Kabat, Chothia, Martin (enhanced Chothia), IMGT and Honegger for light chains and heavy chains. CDR definitions are indicated in orange. Residues impacting affinity and/or angulation are indicated in red. The upper case letter refers to the publication that describes the impact of the residue. These references are listed below: Residues impacting the VH/VL angulation:^(a)^Abhinandan and Martin (35);^(b)^Dunbar et al. (78);^(d)^Chailyan et al. (90), Residues impacting the affinity:^(c)^Chatellier et al. (73);^(e)^Honegger and Plückthun (74); 30 positions impacting the most the free binding energy are indicated in bolt and underlined (5).

## References

[1] Midtvedt K Fauchald P Lien B Hartmann A Albrechtsen D Bjerkely BL. Individualized T cell monitored administration of ATG versus OKT3 in steroid-resistant kidney graft rejection. Clin Transplant. (2003) 17:69–74. 10.1034/j.1399-0012.2003.02105.x 12588325

[2] Smith S. Ten years of Orthoclone OKT3 (muromonab-CD3): a review. J Transpl Coord (1996) 6:109–21. 10.7182/prtr.1.6.3.8145l3u185493182 9188368

[3] Feldmann M Maini RN Anti -TNFα therapy of rheumathoid arthritis: what have we learned? Annu rev Immunol. (2001) 19:163–96. 10.1146/annurev.immunol.19.1.163 11244034

[4] Kunik V Peters B Ofran Y. Structural consensus among antibodies defines the antigen binding site. PLoS Comput Biol. (2012) 8:e1002388. 10.1371/journal.pcbi.1002388 22383868 PMC3285572

[5] Robin G Sato Y Desplancq D Rochel N Weiss E Martineau P. Restricted diversity of antigen binding residues of antibodies revealed by computational alanine scanning of 227 antibody–antigen complexes. J Mol Biol. (2014) 426:3729–3743. 10.1016/j.jmb.2014.08.013 25174334

[6] MacCallum RM Martin a C Thornton JM. Antibody-antigen interactions: contact analysis and binding site topography. J Mol Biol. (1996) 262:732–745. 10.1006/jmbi.1996.0548 8876650

[7] Mitchell LS Colwell LJ. Comparative analysis of nanobody sequence and structure data. Proteins Struct Funct Bioinforma (2018) 86:697–706. 10.1002/prot.25497 29569425 PMC6033041

[8] Nelson AL. Antibody fragments: Hope and hype. MAbs (2010) 2:77–83. 10.4161/mabs.2.1.10786 20093855 PMC2828581

[9] Nuñez-Prado N Compte M Harwood S Álvarez-Méndez A Lykkemark S Sanz L. The coming of age of engineered multivalent antibodies. Drug Discov Today (2015) 20:588–94. 10.1016/j.drudis.2015.02.013 25757598

[10] Vigne E Sassoon I Une liaison réussie entre un anticorps et une petite molécule cytotoxique. La montée en puissance des immunoconjugués en oncologie. Médecine Sci. (2014) 30:855–63. 10.1051/medsci/20143010012 25311020

[11] Guan M Zhou Y-P Sun J-L Chen S-C. Adverse events of monoclonal antibodies used for cancer therapy. Biomed Res Int. (2015) 2015:1–13. 10.1155/2015/428169 26075239 PMC4436450

[12] Mirick GR Bradt BM DeNardo SJ DeNardo GL A review of human anti-globulin antibody (HAGA, HAMA, HACA, HAHA) responses to monoclonal antibodies. Not four letter words. Q J Nucl Med Mol Imaging (2004) 48:251–7. 15640788

[13] DeNardo GL Bradt BM Mirick GR DeNardo SJ. Human antiglobulin response to foreign antibodies: therapeutic benefit? Cancer Immunol Immunother. (2003) 52:309–16. 10.1007/s00262-002-0350-y 12700946 PMC11034264

[14] LoBuglio AF Wheeler RH Trang J Haynes A Rogers K Harvey EB. Mouse/human chimeric monoclonal antibody in man: kinetics and immune response. Proc Natl Acad Sci USA. (1989) 86:4220–4. 10.1073/pnas.86.11.4220 2726771 PMC287422

[15] De Pascalis R Iwahashi M Tamura M Padlan EA Gonzales NR Santos AD Grafting of “Abbreviated” complementarity-determining regions containing specificity-determining residues essential for ligand contact to engineer a less immunogenic humanized monoclonal antibody. J Immunol (2002) 169:3076–84. 10.4049/jimmunol.169.6.3076 12218124

[16] Jones PT Dear PH Foote J Neuberger MS Winter G. Replacing the complementarity-determining regions in a human antibody with those from a mouse. Nature (1986) 321:522–25. 10.1038/321522a0 3713831

[17] Pedersen JT Henry AH Searle SJ Guild BC Roguska M Rees AR. Comparison of surface accessible residues in human and murine immunoglobulin Fv domains. Implication for humanization of murine antibodies. J Mol Biol. (1994) 235:959–73. 10.1006/jmbi.1994.1050 7507176

[18] Padlan EA. A possible procedure for reducing the immunogenicity of antibody variable domains while preserving their ligand-binding properties. Mol Immunol. (1991) 28:489–98. 1905784 10.1016/0161-5890(91)90163-e

[19] Tan P Mitchell D a Buss TN Holmes M a Anasetti C Foote J. “Superhumanized” antibodies: reduction of immunogenic potential by complementarity-determining region grafting with human germline sequences: application to an anti-CD28. J Immunol. (2002) 169:1119–1125. 10.4049/jimmunol.169.2.1119 12097421

[20] Lazar G a Desjarlais JR Jacinto J Karki S Hammond PW. A molecular immunology approach to antibody humanization and functional optimization. Mol Immunol. (2007) 44:1986–98. 10.1016/j.molimm.2006.09.029 17079018

[21] Jespers LS Roberts A Mahler SM Winter G Hoogenboom HR. Guiding the selection of human antibodies from phage display repertoires to a single epitope of an antigen. Biotechnology (1994) 12:899–903. 7521646 10.1038/nbt0994-899

[22] Ahmadzadeh V Farajnia S Feizi MAH Nejad RAK. Antibody humanization methods for development of therapeutic applications. Monoclon Antib Immunodiagn Immunother. (2014) 33:67–73. 10.1089/mab.2013.0080 24746146

[23] Riechmann L Clark M Waldmann H Winter G. Reshaping human antibodies for therapy. Nature (1988) 332:323–7. 10.1038/332323a0 3127726

[24] Sela-Culang I Kunik V Ofran Y. The structural basis of antibody-antigen recognition. Front Immunol. (2013) 4:302. 10.3389/fimmu.2013.00302 24115948 PMC3792396

[25] Wu TT Kabat E a. An analysis of the sequences of the variable regions of Bence Jones proteins and myeloma light chains and their implications for antibody complementarity. J Exp Med. (1970) 132:211–50. 10.1084/jem.132.2.211 5508247 PMC2138737

[26] Kabat EA Wu TT Attempts to locate Complementary-determining residues in the variable positions of light and heavy chains. Ann New York Acad Sci. (1971) 190:382–93. 5290024 10.1111/j.1749-6632.1971.tb13550.x

[27] Capra JD Kehoe JM. Variable region sequences of five human immunoglobulin heavy chains of the VH3 subgroup: definitive identification of four heavy chain hypervariable regions. Proc Natl Acad Sci USA (1974) 71:845–8. 4522793 10.1073/pnas.71.3.845 PMC388111

[28] Kabat EA Wu TT Bilofsky H. Unusual distributions of amino acids in complementarity-determining (hypervariable) segments of heavy and light chains of immunoglobulins and their possible roles in specificity of antibody-combining sites. J Biol Chem. (1977) 252:6609–16. 408353

[29] Kabat EA Te Wu T Bilofsky H (U.S.) NI of H. Sequences of Immunoglobulin Chains: Tabulation Analysis of Amino Acid Sequences of Precursors, V-regions, C-regions, J-Chain BP-Microglobulins, 1979. Department of Health, Education, Welfare, Public Health Service, National Institutes of Health (1979). Available online at: https://books.google.com/books?id=OpW8-ibqyvcC

[30] Kabat EA Te Wu T Foeller C Perry HM Gottesman KS Sequences of Proteins of Immunological Interest. Diane Publishing Company (1992). Available online at: https://books.google.com/books?id=3jMvZYW2ZtwC

[31] Martin AC. Accessing the Kabat antibody sequence database by computer. Proteins (1996) 25:130–3. 10.1002/(SICI)1097-0134(199605)25:1<130::AID-PROT11>3.0.CO;2-L 8727325

[32] Chothia C Lesk a M. Canonical structures for the hypervariable regions of immunoglobulins. J Mol Biol. (1987) 196:901–17. 10.1016/0022-2836(87)90412-8 3681981

[33] Al-Lazikani B Lesk a M Chothia C. Standard conformations for the canonical structures of immunoglobulins. J Mol Biol. (1997) 273:927–48. 10.1006/jmbi.1997.1354 9367782

[34] Chothia C Lesk AM Tramontano A Levitt M Smith-Gill SJ Air G. Conformations of immunoglobulin hypervariable regions. Nature (1989) 342:877–83. 10.1038/342877a0 2687698

[35] Abhinandan KR Martin ACR. Analysis and improvements to Kabat and structurally correct numbering of antibody variable domains. Mol Immunol. (2008) 45:3832–9. 10.1016/j.molimm.2008.05.022 18614234

[36] Gelfand IM Kister a E. Analysis of the relation between the sequence and secondary and three-dimensional structures of immunoglobulin molecules. Proc Natl Acad Sci USA. (1995) 92:10884–8. 10.1073/pnas.92.24.10884 7479903 PMC40535

[37] Gelfand I Kister a Kulikowski C Stoyanov O. Geometric invariant core for the V(L) and V(H) domains of immunoglobulin molecules. Protein Eng. (1998) 11:1015–25. 10.1089/106652701446143 9876922

[38] Gelfand IM Kister a E Leshchiner D. The invariant system of coordinates of antibody molecules: prediction of the “standard” C alpha framework of VL and VH domains. Proc Natl Acad Sci USA. (1996) 93:3675–8. 8622995 10.1073/pnas.93.8.3675 PMC39670

[39] Gelfand I Kister A Kulikowski C Stoyanov O. Algorithmic determination of core positions in the VL and VH domains of immunoglobulin molecules. J Comput Biol. (1998) 5:467–77. 9773343 10.1089/cmb.1998.5.467

[40] Tramontano A Chothia C Lesk AM. Structural determinants of the conformations of medium-sized loops in proteins. Proteins (1989) 6:382–94. 10.1002/prot.340060405 2622909

[41] Giudicelli V Chaume D Bodmer J Müller W Busin C Marsh S. IMGT, the international ImMunoGeneTics database. Nucleic Acids Res. (1997) 25:206–11. 9016537 10.1093/nar/25.1.206 PMC146384

[42] Lefranc MP. Unique database numbering system for immunogenetic analysis. Immunol Today (1997) 18:509. 10.1016/S0167-5699(97)01163-8 9386342

[43] Lefranc M-P Pommié C Ruiz M Giuducelli V Foulquier E Truong L. IMGT unique numbering for immunoglobulin and T cell receptor variable domains and Ig superfamily V-like domains. Dev Comp Immunol. (2003) 27:55–77. 10.1016/S0145-305X(02)00039-3 12477501

[44] Lefranc M-P Giudicelli V Ginestoux C Bosc N Folch G Guiraudou D. IMGT-ONTOLOGY for immunogenetics and immunoinformatics. In Silico Biol. (2004) 4:17–29. 15089751

[45] Ruiz M Giudicelli V Ginestoux C Stoehr P Robinson J Bodmer J. IMGT, the international ImMunoGeneTics database. Nucleic Acids Res. (2000) 28:219–21. 10.1093/nar/28.1.219 10592230 PMC102442

[46] Ehrenmann F Kaas Q Lefranc MP. IMGT/3dstructure-DB and IMGT/domaingapalign: a database and a tool for immunoglobulins or antibodies, T cell receptors, MHC, IgSF and MHcSF. Nucleic Acids Res. (2009) 38:301–7. 10.1093/nar/gkp946 19900967 PMC2808948

[47] Lefranc MP IMGT collier de perles for the variable (V), constant (C), and groove (G) domains of IG, TR, MH, IgSF, and MhSF. Cold Spring Harb Protoc. (2011) 6:643–51. 10.1101/pdb.ip86 21632788

[48] Brochet X Lefranc M-P Giudicelli V. IMGT/V-QUEST: the highly customized and integrated system for IG and TR standardized V-J and V-D-J sequence analysis. Nucleic Acids Res. (2008) 36:W503–8. 10.1093/nar/gkn316 18503082 PMC2447746

[49] Honegger A Plückthun A. Yet another numbering scheme for immunoglobulin variable domains: an automatic modeling and analysis tool. J Mol Biol. (2001) 309:657–70. 10.1006/jmbi.2001.4662 11397087

[50] North B Lehmann A Dunbrack RLJ. A new clustering of antibody CDR loop conformations. J Mol Biol. (2011) 406:228–56. 10.1016/j.jmb.2010.10.030.A 21035459 PMC3065967

[51] Padlan EA. Anatomy of the antibody molecule. Mol Immunol. (1994) 31:169–217. 10.1016/0161-5890(94)90001-9 8114766

[52] Padlan EA Abergel C Tipper JP. Identification of specificity-determining residues in antibodies. FASEB J Off Publ Fed Am Soc Exp Biol. (1995) 9:133–9. 7821752 10.1096/fasebj.9.1.7821752

[53] Nam DH Rodriguez C Remacle AG Strongin AY Ge X. Active-site MMP-selective antibody inhibitors discovered from convex paratope synthetic libraries. Proc Natl Acad Sci USA. (2016) 113:14970–5. 10.1073/pnas.1609375114 27965386 PMC5206513

[54] Stanfield RL Wilson IA Smider VV Biology C Jolla L Jolla L Conservation and diversity in the ultralong third heavy-chain complementarity-determining region of bovine antibodies. Sci Immunol. (2017) 1:1–21. 10.1126/sciimmunol.aaf7962.Conservation PMC5000368 27574710

[55] Ofran Y Schlessinger A Rost B. Automated identification of complementarity determining regions (CDRs) reveals peculiar characteristics of CDRs and B cell epitopes. J Immunol. (2008) 181:6230–5. 10.4049/jimmunol.181.9.6230 18941213

[56] Collis AVJ Brouwer AP Martin ACR. Analysis of the antigen combining site: correlations between length and sequence composition of the hypervariable loops and the nature of the antigen. J Mol Biol. (2003) 325:337–54. 10.1016/S0022-2836(02)01222-6 12488099

[57] Kunik V Ashkenazi S Ofran Y Paratome: an online tool for systematic identification of antigen-binding regions in antibodies based on sequence or structure. Nucleic Acids Res. (2012) 40:521–4. 10.1093/nar/gks480 PMC3394289 22675071

[58] Olimpieri PP Chailyan A Tramontano A Marcatili P. Prediction of site-specific interactions in antibody-antigen complexes: the proABC method and server. Bioinformatics (2013) 29:2285–91. 10.1093/bioinformatics/btt369 23803466 PMC3753563

[59] Jarasch A Skerra A. Aligning, analyzing, and visualizing sequences for antibody engineering: Automated recognition of immunoglobulin variable region features. Proteins Struct Funct Bioinforma (2017) 85:65–71. 10.1002/prot.25193 27770557

[60] Martin ACR Allen J Bioinformatics tools for antibody engineering. In: Dübel S editor. Handbook of Therapeutic Antibodies. Weinheim: Wiley-VCH Verlag GmbH (2008). p. 95–117. 10.1002/9783527619740.ch5

[61] Ewert S Honegger A Pluckthun A. Stability improvement of antibodies for extracellular and intracellular applications: CDR grafting to stable frameworks and structure-based framework engineering. Methods (2004) 34:184–99. 10.1016/j.ymeth.2004.04.007 15312672

[62] Teplyakov A Gilliland GL. Canonical structures of short CDR-L3 in antibodies. Proteins Struct Funct Bioinforma (2014) 82:1668–73. 10.1002/prot.24559 24623659 PMC4260120

[63] Tramontano A Chothia C Lesk AM. Framework residue 71 is a major determinant of the position and conformation of the second hypervariable region in the VH domains of immunoglobulins. J Mol Biol. (1990) 215:175–82. 10.1016/S0022-2836(05)80102-0 2118959

[64] Martin a C Thornton JM. Structural families in loops of homologous proteins: automatic classification, modelling and application to antibodies. J Mol Biol. (1996) 263:800–15. 10.1006/jmbi.1996.0617 8947577

[65] Kuroda D Shirai H Kobori M Nakamura H Systematic classification of CDR-L3 in antibodies: Implications of the light chain subtypes and the V L-V H interface. Proteins Struct Funct Bioinforma (2009) 75:139–46. 10.1002/prot.22230 18798566

[66] Chailyan A Marcatili P Cirillo D Tramontano A. Structural repertoire of immunoglobulin lambda light chains. Proteins (2011) 79:1513–24. 10.1002/prot.22979 21365679

[67] Morea V Tramontano A Rustici M Chothia C Lesk AM. Conformations of the third hypervariable region in the VH domain of immunoglobulins. J Mol Biol. (1998) 275:269–94. 10.1006/jmbi.1997.1442 9466909

[68] Shirai H Kidera A Nakamura H. H3-rules : identification of CDR-H3 structures in antibodies. FEBS Lett. (1999) 455:188–97. 10428499 10.1016/s0014-5793(99)00821-2

[69] Oliva B Bates PA Querol E Aviles FX Sternberg MJ. Automated classification of antibody complementarity determining region 3 of the heavy chain (H3) loops into canonical forms and its application to protein structure prediction. J Mol Biol. (1998) 279:1193–210. 10.1006/jmbi.1998.1847 9642095

[70] De Genst E Handelberg F Van Meirhaeghe A Vynck S Loris R Wyns L Chemical basis for the affinity maturation of a camel single domain antibody. J Biol Chem. (2004) 279:53593–601. 10.1074/jbc.M407843200 15383540

[71] Adolf-Bryfogle J Xu Q North B Lehmann A Dunbrack RL. PyIgClassify: a database of antibody CDR structural classifications. Nucleic Acids Res. (2015) 43:D432–8. 10.1093/nar/gku1106 25392411 PMC4383924

[72] Dunbar J Krawczyk K Leem J Marks C Nowak J Regep C. SAbPred: a structure-based antibody prediction server. Nucleic Acids Res. (2016) 44:W474–8. 10.1093/nar/gkw361 27131379 PMC4987913

[73] Chatellier J Van Regenmortel MH Vernet T Altschuh D. Functional mapping of conserved residues located at the VL and VH domain interface of a Fab. J Mol Biol. (1996) 264:1–6. 10.1006/jmbi.1996.0618 8950262

[74] Honegger A Plückthun A. The influence of the buried glutamine or glutamate residue in position 6 on the structure of immunoglobulin variable domains. J Mol Biol. (2001) 309:687–99. 10.1006/jmbi.2001.4664 11397089

[75] Foote J Winter G. Antibody framework residues affecting the conformation of the hypervariable loops. J Mol Biol. (1992) 224:487–99. 1560463 10.1016/0022-2836(92)91010-m

[76] Chothia C Novotný J Bruccoleri R Karplus M. Domain association in immunoglobulin molecules. The packing of variable domains. J Mol Biol. (1985) 186:651–63. 10.1016/0022-2836(85)90137-8 4093982

[77] Abhinandan KR Martin ACR. Analysis and prediction of VH/VL packing in antibodies. Protein Eng Des Sel. (2010) 23:689–97. 10.1093/protein/gzq043 20591902

[78] Dunbar J Fuchs A Shi J Deane CM. ABangle: Characterising the VH-VL orientation in antibodies. Protein Eng Des Sel. (2013) 26:611–20. 10.1093/protein/gzt020 23708320

[79] Nakanishi T Tsumoto K Yokota A Kondo H Kumagai I. Critical contribution of VH-VL interaction to reshaping of an antibody: the case of humanization of anti-lysozyme antibody, HyHEL-10. Protein Sci. (2008) 17:261–70. 10.1110/ps.073156708 18227432 PMC2222731

[80] Bujotzek A Lipsmeier F Harris SF Benz J Kuglstatter A Georges G. VH-VL orientation prediction for antibody humanization candidate selection: A case study. MAbs (2016) 8:288–305. 10.1080/19420862.2015.1117720 26637054 PMC4966660

[81] Kuroda D Shirai H Kobori M Nakamura H. Structural classification of CDR-H3 revisited: A lesson in antibody modeling. Proteins Struct Funct Genet. (2008) 73:608–20. 10.1002/prot.22087 18473362

[82] Vargas-Madrazo E Paz-García E. An improved model of association for VH-VL immunoglobulin domains: Asymmetries between VH and VL in the packing of some interface residues. J Mol Recognit. (2003) 16:113–20. 10.1002/jmr.613 12833565

[83] Narayana Bhat T Bentley GA Boulot G Greene MI Tello D Dall 'acqua W Bound water molecules and conformational stabilization help mediate an antigen-antibody association (antigen-andbody complex/three-dimensional structure/enthalpy and entropy of association/hydration). Immunology (1994) 91:1089–93. 10.1073/pnas.91.3.1089 PMC521459 8302837

[84] Weitzner BD Kuroda D Marze N Xu J Gray JJ. Blind prediction performance of RosettaAntibody 3.0: Grafting, relaxation, kinematic loop modeling, and full CDR optimization. Proteins Struct Funct Bioinforma (2014) 82:1611–23. 10.1002/prot.24534 24519881 PMC4107143

[85] Liang S Zheng D Zhang C Zacharias M. Prediction of antigenic epitopes on protein surfaces by consensus scoring. BMC Bioinformatics (2009) 10:302. 10.1186/1471-2105-10-302 19772615 PMC2761409

[86] Kulkarni-Kale U Bhosle S Kolaskar AS. CEP: A conformational epitope prediction server. Nucleic Acids Res. (2005) 33:168–71. 10.1093/nar/gki460 15980448 PMC1160221

[87] Sweredoski MJ Baldi P. PEPITO: Improved discontinuous B-cell epitope prediction using multiple distance thresholds and half sphere exposure. Bioinformatics (2008) 24:1459–60. 10.1093/bioinformatics/btn199 18443018

[88] Moise L De Groot A Marcello A Tassone R Martin W Cousens L. Building better biotherapeutics and vaccines by design: EpiVax, Inc., an immunology company. R I Med J. (2013) 96:19–21. 23641421

[89] Seeliger D. Development of Scoring Functions for Antibody Sequence Assessment and Optimization. PLoS ONE (2013) 8:e0076909. 10.1371/journal.pone.0076909 24204701 PMC3804498

[90] Chailyan A Marcatili P Tramontano A. The association of heavy and light chain variable domains in antibodies: implications for antigen specificity. FEBS J. (2011) 278:2858–66. 10.1111/j.1742-4658.2011.08207.x 21651726 PMC3562479

